# The Systematic Classification of Gallbladder Stones

**DOI:** 10.1371/journal.pone.0074887

**Published:** 2013-10-04

**Authors:** Tie Qiao, Rui-hong Ma, Xiao-bing Luo, Liu-qing Yang, Zhen-liang Luo, Pei-ming Zheng

**Affiliations:** 1 Laboratory of Gallbladder Diseases, Institute of Gallbladder Disease of Panyu, Panyu, Guangzhou, People's Republic of China; 2 Laboratory of Gallbladder Diseases, The Sixth People's Hospital of Nansha, Nansha, Guangzhou, People's Republic of China; National Institute for Viral Disease Control and Prevention, CDC, China, China

## Abstract

**Background:**

To develop a method for systematic classification of gallbladder stones, analyze the clinical characteristics of each type of stone and provide a theoretical basis for the study of the formation mechanism of different types of gallbladder stones.

**Methodology:**

A total of 807 consecutive patients with gallbladder stones were enrolled and their gallstones were studied. The material composition of gallbladder stones was analyzed using Fourier Transform Infrared spectroscopy and the distribution and microstructure of material components was observed with Scanning Electron Microscopy. The composition and distribution of elements were analyzed by an X-ray energy spectrometer. Gallbladder stones were classified accordingly, and then, gender, age, medical history and BMI of patients with each type of stone were analyzed.

**Principal Findings:**

Gallbladder stones were classified into 8 types and more than ten subtypes, including cholesterol stones (297), pigment stones (217), calcium carbonate stones (139), phosphate stones (12), calcium stearate stones (9), protein stones (3), cystine stones (1) and mixed stones (129). Mixed stones were those stones with two or more than two kinds of material components and the content of each component was similar. A total of 11 subtypes of mixed stones were found in this study. Patients with cholesterol stones were mainly female between the ages of 30 and 50, with higher BMI and shorter medical history than patients with pigment stones (*P<0.05*), however, patients with pigment, calcium carbonate, phosphate stones were mainly male between the ages of 40 and 60.

**Conclusion:**

The systematic classification of gallbladder stones indicates that different types of stones have different characteristics in terms of the microstructure, elemental composition and distribution, providing an important basis for the mechanistic study of gallbladder stones.

## Introduction

Cholecystolithiasis is a common disease worldwide [1]–[4]. The incidence of gallstones is 15% in America, 5.9∼21.9% in Europe, 4∼15% in Asia and 3∼11% in China [5]–[7]. Current research suggests that different types of gallstones have different pathogenesis [8]–[16]. Research on the systematic classification of gallbladder stones may help to reveal the formation mechanism of different types of gallstones. The traditional classification scheme classified gallstones into 3 types according to cholesterol content, including cholesterol stone (cholesterol content ≥70%), pigment stone (cholesterol content ≤30%) and mixed stone (30% ≤cholesterol content ≤70%) [17]. Professor Fu et al (1984) divided gallstones into 8 types according to the profile structure and chemical components. These included radial, radial annual ring-like, rock strata-like stromatolite, cast amorphous, sand bed-like stromatolite, silt-like, black, and complex stones [18]. Among these, the radial, radial annual ring-like, and rock strata-like stromatolite stones were cholesterol stones, and the cast amorphous, sand bed-like stromatolite, and silt-like stones were pigment stones. With the application of infrared spectroscopy in recent years gallstones have been classified into cholesterol stones, pigment stones, mixed stones and other rare stones (including calcium carbonate, calcium phosphate, and fatty acid calcium stones) [19], [20]. The traditional classification, using one or two means (chemical components, the profile structure and chemical components or infrared spectroscopy), is rough and not very accurate, therefore, we question the traditional classification for gallbladder stones and raise the possibility of developing a systematic classification scheme. Using FTIR Spectroscopy, Scanning Electron Microscopy and X-ray energy spectrometer and classifying gallstones according to the appearance, profile structure, component content and distribution, microstructure, elemental composition and distribution, the new systematic classification scheme, makes up the deficiency of the traditional classification, and thus is more accurate.

## Materials and Methods

### Ethics Statement

A written informed consent was obtained from all subjects. This research was approved by the Medical Ethics Committee of The Sixth People's Hospital of Nansha, Guangzhou. A written informed consent was obtained from the guardians on the behalf of the minors/children participants involved in our study.

### Subjects and Specimens

Gallbladder stones from 807 consecutive cholecystolithiasis patients that received gallbladder-preserving cholelithotomies [21] in the Department of General Surgery of The Sixth People's Hospital of Nansha, Guangzhou, between January 2009 and November 2012, were studied. The patients consisted of 423 men, ranging in age from 17 to 77 (mean  = 45.39±11.87) years old and 384 women, ranging in age from 10 to 80 (mean  = 46.07±12.89) years old. There was no significant difference when comparing the age of the two groups. Gallbladder stone from the same one patient was deemed as one case. All the stones were washed twice with distilled water, and then dried.

### Composition Analysis of Gallbladder Stones by FTIR Spectroscopy

A total of 807 gallbladder stones were analyzed using FTIR spectroscopy. A 2 mg sample of each layer was weighed if the layered structures were distinct. In amorphous stones 2 mg samples were weighed directly. The samples were mixed with KBr at a ratio of 1∶150, ground thoroughly, and used to make discs. The main components were analyzed using a Bruker (TENSOR27, Germany) FTIR spectrometer in the frequency range of 400–4000 cm^−1^, at 4 cm^−1^ resolution. Control substances (99% pure standard) were obtained from Sigma Chemical Company (St. Louis, MO, USA). Gallstone composition was determined by comparison of the gallbladder stones with standard control spectra [22], [23].

The distribution of material components and microstructure was observed using Scanning Electron Microscopy and the elemental composition and distribution was analyzed by an X-ray energy spectrometer.

A total of 240 gallbladder stones, including those stones not identified by FTIR Spectroscopy, were randomly selected for SEM analysis. The stones were split and 1–2 pieces (3–5 mm in size) were sampled from each layer if the layered structures were distinct. In amorphous stones, 1–2 pieces, 3–5 mm in size, were sampled and fixed on the sample table using an electro-conductive adhesive. One piece was secured to the surface while the other was placed facing the opposite direction. The surface to be analyzed was polished, thus making the surface and the bottom surface parallel. The samples were then dried at 60°C overnight. The dried samples were subsequently sputter-coated with gold (ETD-2000, Beijing Elaborate Technology Development Ltd., China) and observed using a ZEISS (EVO LS10, Cambridge, Germany) SEM and photographed. The EHT was 20 kv. The region of interest was amplified to 400 and the composition and distribution of elements was analyzed by X-ray energy spectrometer (X-Max, OXFORD, England). The microstructure was then analyzed through amplification of 1000, 3000, 6000, 10000, and 20000. The elements from B5 to U92 could be detected as long as their weight percentage was more than 0.01%. The microstructure of each component was determined according to FTIR Spectroscopy, the X-ray energy spectrometer as well as SEM, and the content and distribution of each component was analyzed using SEM.

### Identification of Stone type

Gallbladder stones were classified according to the appearance, profile structure, component content and distribution, microstructure, elemental composition and distribution: if the layered structures were distinct, the stone type was identified using FTIR Spectroscopy. For amorphous stones, samples from both the surface and the nucleus were analyzed via FTIR Spectroscopy. The stone type could be identified if the 2 samples were consistent, otherwise, the stones were classified according to the content and distribution of the component found using SEM. If there were two or more than two kinds of material components and the content of the each component was similar, the stone was classified as a mixed stone. If there was only one main component, the stone was classified as a simple stone, such as cholesterol, pigment, calcium carbonate, phosphate, calcium stearate, protein, or cystine.

### Statistical analysis

Age and medical history were analyzed using One-Way ANOVA and presented as mean ± SD, while the ratio of male and female, as well as the ratio of patients with normal weight and overweight were analyzed using a chi square test. LSD and the partitions of the chi square method were used for multiple comparisons using *SPSS v.11.5* software. *P<0.05* was regarded as statistically significant.

## Results

1. The appearance characteristics and the infrared spectrogram of each stone type

**Figure 1:**
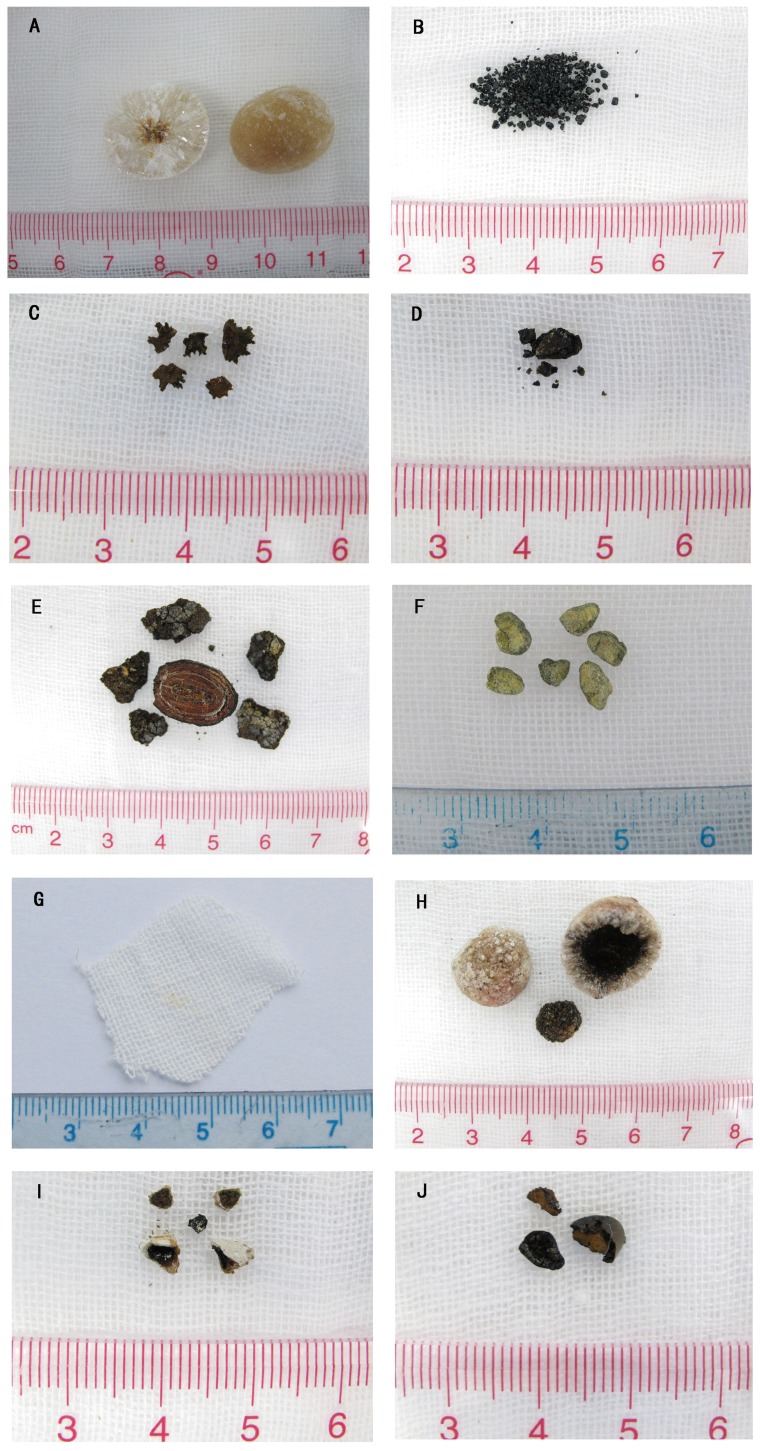
The appearance of each type of gallbladder stone. A. Cholesterol stone B. Pigment stone C. Calcium carbonate stone D. Phosphate stone E. Calcium stearate stone F. Protein stone G. Cystine stone H. Cholesterol- bilirubinate mixed stone I. Bilirubinate -calcium carbonate mixed stone J. Bilirubinate -phosphate mixed stone.

**Figure 2:**
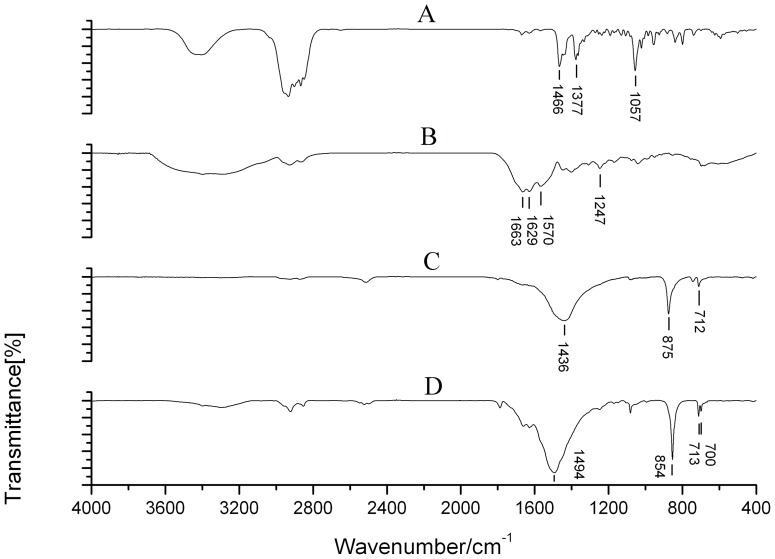
A typical IR spectrogram of the three most common gallbladder stone types. A. Cholesterol stone: 1466, 1377, and 1057 cm^−1^ were the characteristic absorption peaks of cholesterol. B. Pigment stone: 1663, 1629, 1570, and 1247 cm^−1^ were the characteristic absorption peaks of bilirubin and bilirubinate. C. Calcium carbonate of calcite type: 1436, 875, and 712 cm^−1^ were the characteristic absorption peaks of calcium carbonate of calcite type. D. Calcium carbonate of aragonite type: 1494, 854, 713, and 700 cm^−1^ were the characteristic absorption peaks of calcium carbonate of aragonite type.

**Figure 3:**
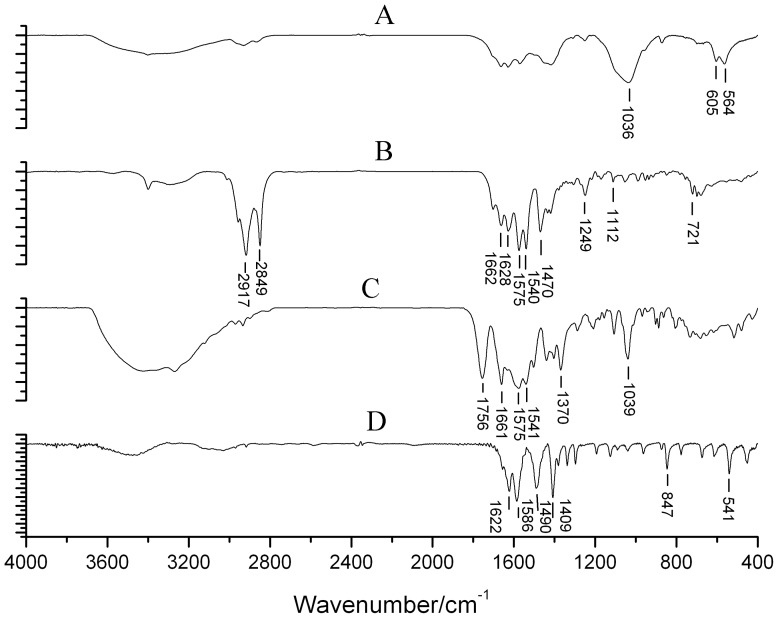
A typical IR spectrogram of four rare gallbladder stone types. A. Phosphate stone: 1036, 605, 564 were the characteristic absorption peaks of phosphate. B. Calcium stearate stone: 2917, 2849, 1575, 1540, 1470, 1112, and 721 cm^−1^ were the characteristic absorption peaks of calcium stearate. C. protein stone: 1756, 1661, 1575, 1541, 1370, and 1039 cm^−1^ were the absorption peaks of protein. D. cystine stone: 1622, 1566, 1490, 1409, 847, and 541 cm^−1^ were the absorption peaks of cystine.

**Figure 4:**
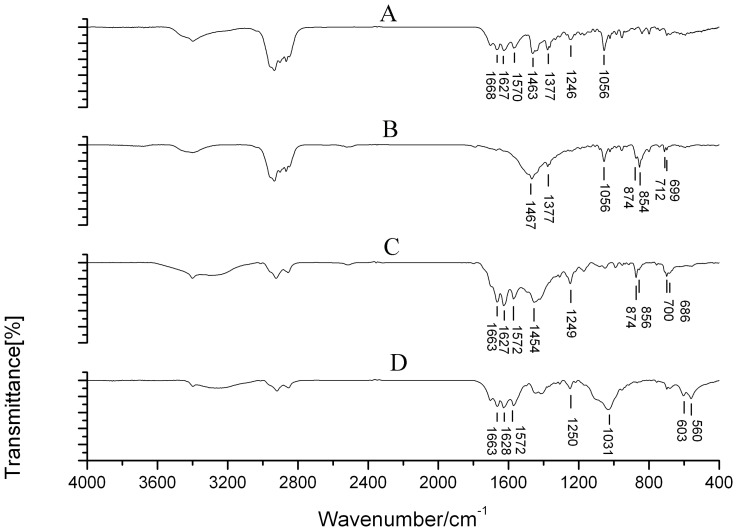
A typical IR spectrogram of four common mixed gallbladder stone types. A. Cholesterol- bilirubinate mixed stone: 1666, 1570, and 1246 cm^−1^ were the absorption peaks of bilirubin and bilirubinate, while 1463, 1377, and 1056 cm^−1^ were those of cholesterol. B. Cholesterol- calcium carbonate mixed stone: 1467, 1377 and 1056 cm^−1^ were the absorption peaks of cholesterol, while 1467, 874, 854, 712, 699 were those of calcium carbonate, and 1467 was the fusion peak of cholesterol and calcium carbonate. C. Bilirubinate-calcium carbonate mixed stone: 1663, 1572, and 1249 cm^−1^ were the absorption peaks of bilirubin and bilirubinate, while 1454, 874, 856, 700 and 686 cm^−1^ were those of calcium carbonate. D. Bilirubinate-phosphate mixed stone: 1663, 1572, and 1250 cm^−1^ were the absorption peaks of bilirubin and bilirubinate, while 1031, 603, and 560 cm^−1^ were those of phosphate.

Gallbladder stones were classified into 8 types and more than ten subtypes according to the systematic classification. These included cholesterol stones, pigment stones, calcium carbonate stones, phosphate stones, calcium stearate stones, protein stones, cystine stones and mixed stones (Table 1). Cholesterol stones appeared brownish yellow, amber, grey, celadon or black and were spherical or polyhedron in shape. They were of different sizes, soft, and the surfaces were smooth and glossy or rough. The profile was yellow, brownish yellow or white with a radial or radial armillary layered arrangement and/or a darker nucleus, with no distinct layer (Figure 1A). Pigment stones were amorphous, brittle, granules that were black, charcoal grey or grayish brown in color, with no layer in the profile (Figure 1B). Calcium carbonate stones were black coralline-like, green mud-like or black amorphous granules, hard or brittle in texture, and had no layer in the profile (Figure 1C). Phosphate stones were smooth surfaced and brittle with a black coal cinder-like color (Figure 1D). Calcium stearate stones were brittle and irregular granules, brick red or grey in color, and with a brick-red profile with no layer, (Figure 1E). Protein stones were hard in texture, green in color, with clay or mud shapes, and no layer in the profile (Figure 1F). Cystine stones, a new type of gallbladder stone discovered in our previous studies [24], were tiny, amber particles less than 1 mm in size (Figure 1G). Mixed stones were those stones with two or more than two kinds of material components and the content of each component was similar. There were more than 10 subtypes of mixed stones, 11 of which were found in this study. These subtypes included cholesterol- bilirubinate, bilirubinate -calcium carbonate, cholesterol- calcium carbonate, bilirubinate -phosphate, cholesterol-phosphate, bilirubinate-calcium stearate, calcium carbonate-phosphate, cholesterol- bilirubinate -calcium carbonate, bilirubinate -calcium carbonate- phosphate, cholesterol- bilirubinate -phosphate and cholesterol- calcium carbonate- calcium stearate mixed stones. The first four subtypes were most common. The proportion of each subtype is shown in table 2. Most mixed stones were black, gray-black, brownish yellow, green, celadon or ivory, were large (>1 cm) and spherical in shape. The layered structure of the profile was clear (Figure 1 H–J). Some were amorphous, black or charcoal grey granules, with no layered structure in the profile. The texture was different depending on the mixed components. The infrared spectrogram of each stone type is shown in figures 2–4.

**Table 1:** Clinical characteristics of patients with each type of stone.

Stone Type			Cholesterol	Pigment	Calcium carbonate	Phosphate	Calcium stearate	Protein	Cystine	Mixed	Total
**No**			297	217	139	12	9	3	1	129	807
**Rate (%)**			36.8	26.9	17.2	1.5	1.1	0.4	0.1	16	100
**Age (years) & Gender**	**Male**	**<20**	2	1	0	0	0	0	0	0	3
		**20∼29**	13	8	3	0	0	0	0	6	30
		**30∼39**	55	28	13	1	0	1	0	11	109
		**40∼49**	39	42	36	3	0	1	0	18	139
		**50∼59**	11	27	19	3	1	0	0	20	81
		**60∼69**	9	16	13	2	1	0	0	9	50
		**≥70**	3	3	2	0	0	0	0	3	11
		**Total**	132	125	86	9	2	2	0	67	423
	**Femal**e	**<20**	2	0	0	1	0	0	0	0	3
		**20∼29**	18	5	2	0	0	1	0	6	32
		**30∼39**	61	15	13	0	0	0	1	14	104
		**40∼49**	45	12	18	0	2	0	0	16	93
		**50∼59**	19	34	11	0	2	0	0	17	83
		**60∼69**	16	24	6	2	1	0	0	8	57
		**≥70**	4	2	3	0	2	0	0	1	12
		**Total**	165	92	53	3	7	1	1	62	384
	**Age average**		41.6±11.9	48. 6±12.0	47.9±10.9	49.5±16.0	58. 6±11.6	34.0±11.5	38	47.0±12.6	45.7±12.4
**Medical history (years)**	**Range**	**≤1**	166	99	84	6	4	3	1	71	434
		**1∼5**	86	65	29	2	5	0	0	33	220
		**>5**	45	53	26	4	0	0	0	25	153
	**Average**		2. 6±4.0	3.4±4.6	2.7±4.4	4.8±7.3	1.5±1.2	0.1±0.0	0.1	2.7±3.5	2.8±4.2
**BMI**	**Range**	**<24**	162	158	77	7	9	3	1	78	495
		**≥24**	135	59	62	5	0	0	0	51	312
	**Average**		23.7±3.5	22.3±3.4	23.7±3.4	22.3±4.6	20.6±2.5	21.4±0.1	20.3	23.2±3.7	23.2±3.5

**Table 2:** Proportion of different subtypes of mixed stones.

Subtype	No.	Rate
Cholesterol- bilirubinate mixed	30	23.3%
Bilirubinate -calcium carbonate mixed	44	34.1%
Cholesterol- calcium carbonate mixed	21	16.3%
Bilirubinate -phosphate mixed	14	10.9%
Cholesterol-phosphate mixed	4	3.1%
Bilirubinate-calcium stearate mixed	2	1.6%
Calcium carbonate-phosphate mixed	2	1.6%
Cholesterol- bilirubinate -calcium carbonate mixed	6	4.7%
Bilirubinate -calcium carbonate- phosphate mixed	3	2.3%
Cholesterol- bilirubinate -phosphate mixed	2	1.6%
Cholesterol- calcium carbonate- calcium stearate mixed	1	0.8%
Total	129	100.0%

2. Microstructure of each type of gallbladder stone

**Figure 5:**
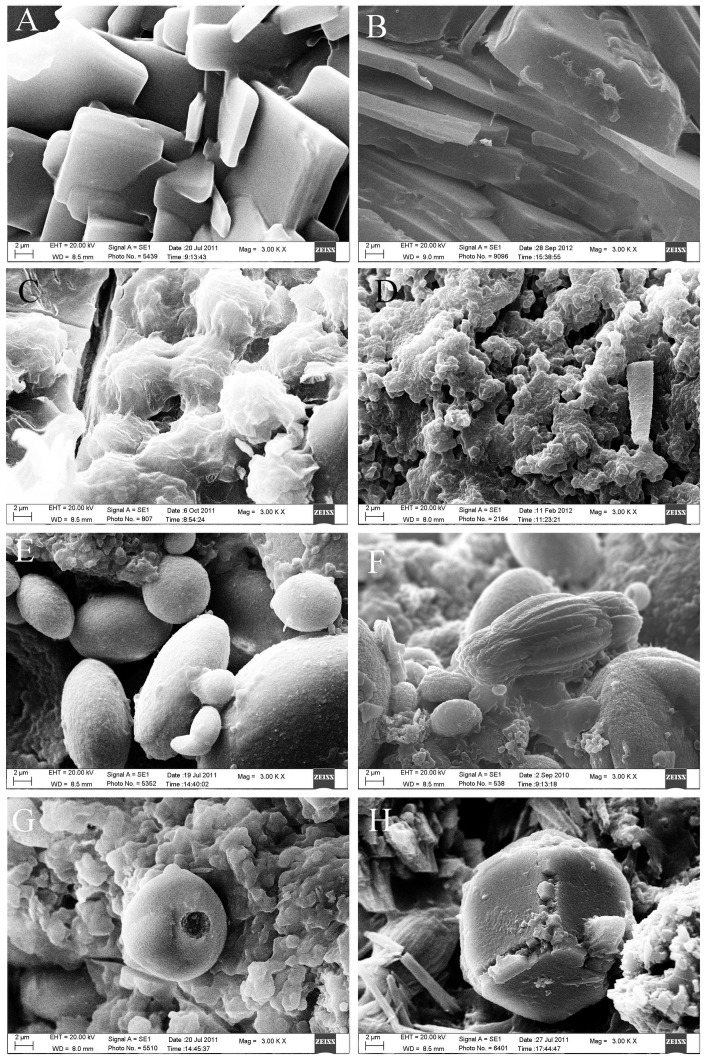
Microstructure of the three most common gallbladder stone types (original magnification ×3000). A, B. Cholesterol stone. A. Plate-like cholesterol crystals. B. Lamellar cholesterol crystals. C, D. Pigment stone. C. Clumping-like bilirubinate particles. D. Irregular bilirubinate particles. E – H. Calcium carbonate stone. E. Bulbiform and ellipsoid calcium carbonate crystals. F. Fusiform calcium carbonate crystal. G. Hawthorn-like calcium carbonate crystal. H. Cuboid calcium carbonate crystal.

**Figure 6:**
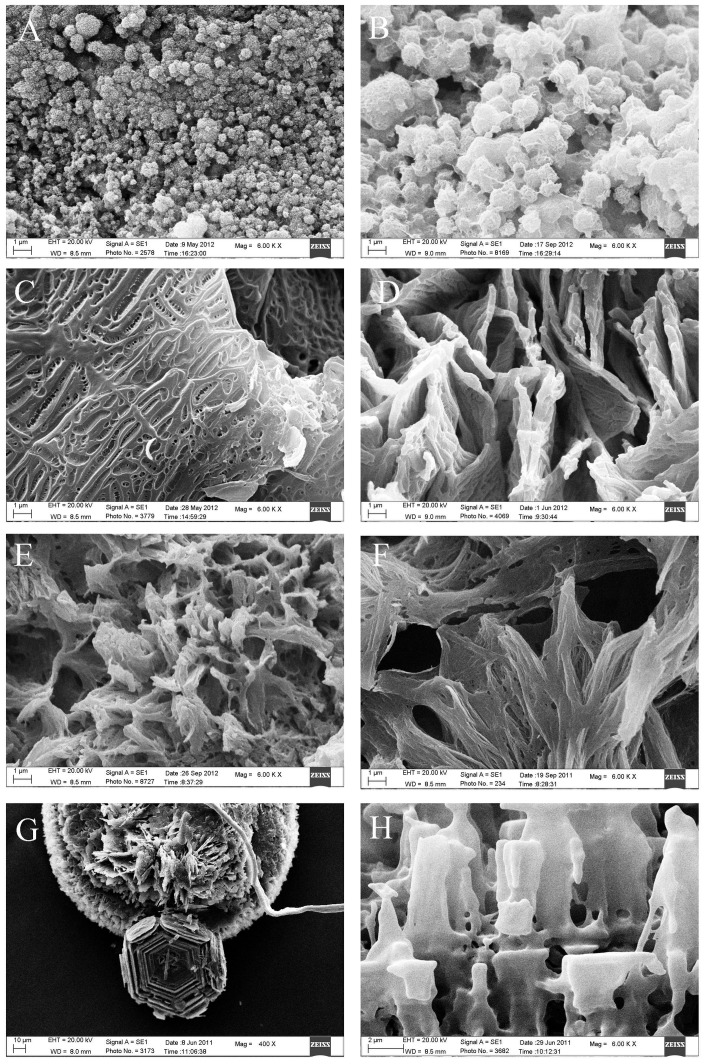
Microstructure of four rare gallbladder stone types. A, B. Phosphate stone (original magnification ×6000). A. Echin-sphere phosphate particles. B. Rough bulbiform phosphate particles. C,D. Calcium stearate stone (original magnification ×6000). C. Calcium stearate presented as network structure. D. Calcium stearate presented as staggered arranged network, protein stone (original magnification×6000). E. Coralliform protein and bilirubinate complex. F. Chrysanthemum petal-like protein and bilirubinate complex. G, H. Cystine stone. G. Stacked hexagonal crystals, some with prominent edges (original magnification ×400). H. The prominent edges appeared lamellar after magnification (original magnification ×6000).

**Figure 7:**
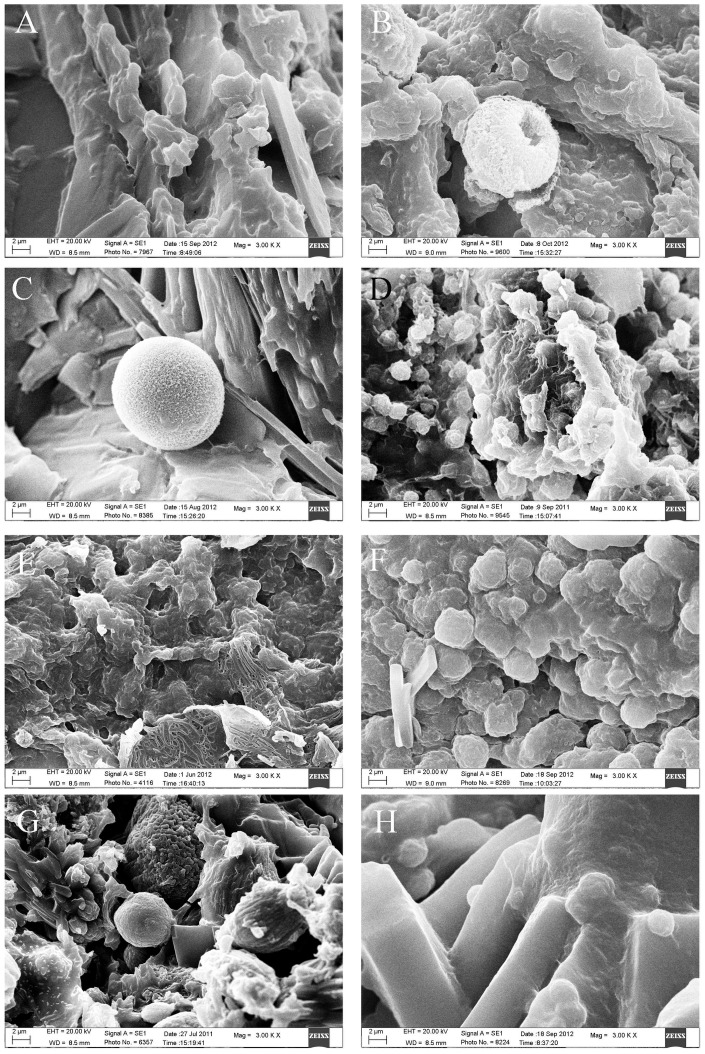
Microstructure of mixed stones (original magnification ×3000). A. Cholesterol- bilirubinate mixed crystals. B. Bilirubinate- calcium carbonate mixed crystals. C. Cholesterol- calcium carbonate mixed crystals. D. Bilirubinate- phosphate mixed particles. E. Bilirubinate- calcium stearate mixed particles. F. Cholesterol- phosphate mixed crystals. G. Cholesterol- bilirubinate- calcium carbonate mixed crystals. H. Cholesterol- bilirubinate- phosphate mixed crystals.

SEM revealed that the cholesterol stones were mainly composed of plate-like or lamellar, tightly stacked cholesterol crystals with a radial, cordlike, irregular, or staggered arrangement. A small amount of bilirubinate particles were occasionally seen. Pigment stones were mainly composed of different sized sphere-like, clumping-like or irregular bilirubinate particles which were loosely arranged. Calcium carbonate stones were composed of various calcium carbonate crystals, with more than ten shapes. Crystal shapes were most commonly bulbiform, but ellipsoid, fagot-like, fusiform, hawthorn-like, cuboid, button, lamellar, broken firewood-like, rod and acicular shapes were also observed (Figure 5). Phosphate stones were mainly composed of echin-sphere or rough bulbiform crystals of different sizes adhered to bilirubinate particles. Calcium stearate stones commonly had a network structure with adherent bilirubinate particles. Protein stones frequently had a honeycomb or chrysanthemum petal structure with adherent bilirubinate particles. Cystine stones were composed of hexagonal cystine crystals, some with prominences along the edges (Figure 6). The shape and the microstructure of mixed stones under SEM varied depending on the different mixed components. Cholesterol- bilirubinate mixed stones were mainly composed of plate-like or lamellar cholesterol crystals and irregular bilirubinate particles. Bilirubinate -calcium carbonate mixed stones were mainly composed of irregular bilirubinate particles with many shapes of calcium carbonate crystals. Cholesterol- calcium carbonate mixed stones were mainly composed of plate-like or lamellar cholesterol crystals with many shapes of calcium carbonate crystals. Bilirubinate -phosphate mixed stones were mainly composed of irregular bilirubinate particles and phosphate particles (Figure 7).

3. The elements composition and distribution of each type of stone

The main elemental composition of cholesterol stones was carbon and oxygen along with very small amounts of other elements, such as calcium, sodium, chlorine, aluminum, magnesium, copper, silicon. Calcium was mainly concentrated in the core, and was mostly in the form of calcium bilirubinate, calcium stearate or calcium carbonate; calcium was rarely in the periphery of the stone. A thin layer of calcium carbonate covered surface of a small number of cholesterol stones. In these stones, calcium was concentrated both in the core and at the surface. The elemental composition of the pigment stones was complicated. The major elements were carbon and oxygen along with more than 10 kinds of mineral elements. The calcium content was significantly higher than that in the cholesterol stones, and was mostly in the form of calcium bilirubinate. Calcium was generally distributed in the stone, and was sometimes in the form of calcium carbonate or calcium stearate, which was mainly distributed in the core and surface. The main elemental composition of the calcium carbonate stones was carbon, oxygen, and calcium, the proportion of which was different than that in the calcium carbonate compound since the calcium carbonate crystals were often adhered to bilirubinate. There were irregular calcium carbonate crystals wrapped with mucoid material or bilirubinate on the surface of the calcium carbonate stones, and many shapes of calcium carbonate crystals such as bulbiform, ellipsoid, fusiform, hawthorn-like, cuboid, button, lamellar, broken firewood-like, rod and acicular in the periphery and core of the profile. The main elemental composition of phosphate stones was carbon, oxygen, calcium and phosphorus, along with a small amount of other elements. There were phosphate particles wrapped with bilirubinate on the surface, with echin-sphere or rough bulbiform crystals adhered to a small number of bilirubinate particles in the periphery and core of the profile. In some cases cholesterol crystals could be seen on the surface and in the core. The main elemental composition of calcium stearate stones was carbon, oxygen and calcium. These stones often contained sulphur, copper and a small amount of other mineral elements. There was bilirubinate on the surface of these stones, and they had a network structure with adherent bilirubinate particles or a small amount of cholesterol crystals in the periphery and core of the profile. The main elemental composition of protein stones was carbon, oxygen, nitrogen, sulphur and calcium, along with a small amount of other elements. There was bilirubinate, protein and/or a few calcium carbonate crystals on the surface, and a complex of protein and bilirubinate mixed with a few calcium carbonate crystals in the periphery and core of the profile. The main elemental composition of cystine stones was carbon, oxygen, nitrogen and sulphur, together with a small quantity of other elements. The elemental composition and content of mixed stones varied with the mixed components (Table 3).

**Table 3:** Elemental composition of each type of stone.

Stone type	Elements composition (%)
Cholesterol	C 82.36±1.76, O 15.64±1.39, a small amount of Ca, Na, Cl, Al, Mg, F, Si
Pigment	C 63.01±3.58, O 28.95±2.26, Ca 5.30±1.99, many kinds of mineral elements, such as Na, Mg, Al, Cu, S, Fe, Zn, K, Cl, F, Mn, Nb
Calcium carbonate	C 32.93±8.6, O 38.38±4.25, Ca 27.75±5.88, a small amount of Na, Mg, Al, Cu, Si, K, Fe
Phosphate	C 44.63±10.23, O 30.46±6.24, Ca 17.37±3.84, P 6.68±2.04, a small amount of Na, Mg, Al, Cu, Fe, Mn, F, Cl, Nb
Calcium stearate	C 69.22±2.19, O 20.93±0.66, Ca 753±1.80, a small amount of S, Cu, Zn, Na, Mg, Al, Cl, F
Protein	C 48.83±7.78, O 24.13±3.48, N 6.16±2.72, S 13.69±2.40, Ca 9.75±2.75, a small amount of Na, Mg, Al, Cl, Si
Cystine	C 50.31±7.60, O 10.32±4.54, N 14.32±2.60, S 30.81±9.31, a small amount of Ca, Na, Mg, Si, Cl
Mixed	varied with the mixed components

4. The clinical characteristics of each type of stone

The gender, age, medical history and BMI of patients with each type of stone were shown in table 1. The proportion of female patients in cholesterol and calcium stearate stones was higher than that in pigment, calcium carbonate and phosphate stones (*P<0.05*), while the proportion of male patients in pigment, calcium carbonate and phosphate stones was higher than that in cholesterol and calcium stearate stones (*P<0.05*). Patients with cholesterol stones were between the ages of 30 and 50 and mainly female; patients with pigment stones were mainly male between the ages of 30 and 60 and female between the ages of 50 and 70; patients with calcium carbonate and phosphate stones were mainly male between ages of 40 and 60; patients with calcium stearate stones were mainly female over the ages of 40, patients with mixed stones were between the ages of 30 and 60 (both male and female). The ages of patients with pigment, calcium carbonate, phosphate or calcium stearate stones were older than those with cholesterol or protein stones (*P<0.05*). The medical history of pigment stones was longer than that of cholesterol stones (*P<0.05*), and the proportion of patients with pigment stones that had long medical history (>5 years) was higher than that with cholesterol stones (Table 1). The BMI of patients with cholesterol stones was higher than that with pigment and calcium stearate stones (*P<0.05*), however, the BMI of patients with pigment and calcium stearate stones was lower than that with cholesterol, calcium carbonate and mixed stones (*P<0.05*). The proportion of overweight patients in cholesterol stones was higher than that in pigment stones and calcium stearate stones (*P<0.05*), the proportion of overweight patients in pigment stones was lower than that in cholesterol, calcium carbonate and mixed stones (*P<0.05*) (Table 1).

## Discussion

In the traditional classification scheme gallstones were grouped into 3 types according to cholesterol content. This included cholesterol stones, pigment stones and mixed stones [17], however, this classification scheme is rough and not very accurate. According to the systematic classification presented in the current study, stones with cholesterol content ≤30% include not only pigment stones, but also calcium carbonate stones, phosphate stones, calcium stearate stones, protein stones, cystine stones, as well as some subtypes of mixed stones such as bilirubinate -calcium carbonate, bilirubinate-phosphate, bilirubinate-calcium stearate, calcium carbonate-phosphate, and bilirubinate -calcium carbonate- phosphate. The traditional classification scheme ignored the above stone types and subtypes. Fu et al. divided gallstones into 8 types according to the profile structure and chemical components [18], discovering and summarizing the relationship between the profile structure and chemical components. The chemical components could be judged roughly through the profile structure, which was suitable for clinical application. According to this classification scheme, cast amorphous, sand bed-like stromatolite, and silt-like stones were pigment stones, however, many stone types such as calcium carbonate, phosphate, calcium stearate, protein and cystine as well as some subtypes of mixed stones may present as cast amorphous, sand bed-like stromatolite, or silt-like, so the classification scheme of Fu et al. may not be precise enough.

With the application of infrared spectroscopy, gallstone classification based on component analysis has been gradually recognized and widely used [25]–[29]. Using FTIR spectroscopy to identify stone composition has many advantages. It is fast, highly sensitive repeatable, and only requires a small sample, and thus, it is one of the best ways to analyze stone composition. Through analysis of the position, shape and intensity of the absorption peak of the infrared spectrum (IRS), we can examine the test samples qualitatively and quantitatively and infer the structure of the unknown compound. Therefore the IR spectrum has become known as a “fingerprint” of the compound [30]. Previous research has indicated that FTIR analysis provides important information regarding gallstone composition and may be an effective tool for research into the mechanism of gallstone formation [20]. However, because the sample requirement is so small (1∼2 mg), FTIR analysis is not accurate for complicated stones especially those amorphous stones with heterogeneous distribution. Protein stones may be judged as pigment stone through FTIR analysis because of the overlap of vibrational bands of the amide and calcium bilirubinate [31], but the two types of stones can be identified by element analysis with an X-ray energy spectrometer. In the present study, the material composition of gallbladder stones was analyzed using Fourier Transform Infrared spectroscopy, the distribution of material components and the microstructure was observed with Scanning Electron Microscopy and the elemental composition and distribution was analyzed by an X-ray energy spectrometer. This combination makes the systematic classification that used to judge stone type more accurately, and makes up for the deficiency of FTIR analysis.

Using systematic classification in the present study, we found 8 types and 11 subtypes of stones, and the appearance, profile structure, microstructure, elemental composition and distribution were analyzed. The appearance, profile structure and texture of some types of stones were similar. For example, most pigment stones, some calcium carbonate stones, phosphate stones and some amorphous mixed stones can't be identified using the traditional classification scheme, however, FTIR spectroscopy revealed that the material components was different, SEM revealed that the microstructure and morphology was different and the X-ray energy spectrometer revealed that the elemental composition and distribution was also different when comparing these stones. In the present study, FTIR Spectroscopy and the X-ray energy spectrometer were used to determine the material components and SEM was used to determine the microstructure of material components as well as the content and distribution of components; this was very helpful in judging the stone type.

Gallbladder stone disease is a complex disease which may be affected by a complex interaction of environmental and genetic factors [15], [32]–[34], so the stone type and proportion have regional differences. Most of the patients in the present study were from Guangdong and Guangxi – two clonorchiasis endemic areas in China. Since *Clonorchis Sinensis* infection was associated with the formation of pigment stones [35], the proportion of pigment stone in the present study was high. In contrast, cholesterol gallstones were predominant in developed countries and some developing countries such as Saudi Arabia [6], [36]. In addition, the proportion of calcium carbonate stone was 17.2%, which was similar to that in Taiwan (14%) [37], yet according to the previous report, calcium carbonate stone was a rare stone type, and mainly occurred in children [38], [39]. The differences may due to the environmental and genetic factors, as Taiwan is also a high incident region of clonorchiasis. But the definite cause still needs further research. Cholesterol stone formation was associated with cholesterol supersaturation, nucleation and precipitation and gallbladder hypomotility, as well as the abnormal expression of the related genes such as ATP binding cassette G5/G8 (ABCG5/G8), liver X receptor α (LXRα), scavenger receptor B type I (SRB1), Niemann Pick C1 like 1 (NPC1L1), mucin genes and CCKR [40]–[42]. Pigment stone formation was associated with bacterial and parasitic infections, glucoprotein and free radical [10], [11], [35], [43]. In the present study, the data indicated that patients having cholesterol stones were mainly female between the ages of 30 and 50, and with higher BMI, which was consistent with previous research [6]. Patients having pigment, calcium carbonate, phosphate and calcium stearate stones had different clinical characteristics compared with those having cholesterol stones, which suggests that the stone formation may be different.

The results of the present study showed that gallbladder stones can be classified into 8 types, and patients with different types of stones have different clinical characteristics. Moreover, it suggests different types of stone may have different formation mechanism. Therefore, for patients with cholelithiasis, their gallstones should be analyzed and classified accurately after surgery. It has great significance not only for individual therapy and prevention, but also for analyzing the distribution of different stone types among different areas and making corresponding preventative strategies. Furthermore, it may provide important data for further research into gallbladder stone formation.

## Conclusion

Accurate classification is a prerequisite for gallstone formation research. The present study provides a new systematic classification for gallstones, which is more accurate compared with the traditional classification. The structure and composition of gallstones provides information about the process of stone formation. The present research suggests that different types of gallbladder stones have characteristic microstructures and morphologies, as well as different elemental composition and distribution, indicating that the formation mechanism of different types of gallbladder stones is not the same. This work provides an objective basis for further research into gallbladder stone formation, meanwhile, it has great significance in the treatment and prevention of gallbladder stones.
